# Olverembatinib combined with inotuzumab ozogamicin in relapsed refractory Philadelphia chromosome-positive acute lymphoblastic leukemia: A case report

**DOI:** 10.1097/MD.0000000000038985

**Published:** 2024-07-19

**Authors:** Tong Liu, Chang Wang, Yu Fu, Yan-ping Yang, Ye-hui Tan

**Affiliations:** a Department of Hematology, The First Hospital of Jilin University, Changchun, China; b Department of Oncology, The First Hospital of Jilin University, Changchun, China.

**Keywords:** inotuzumab ozogamicin, olverembatinib, Philadelphia chromosome-positive acute lymphoblastic leukemia, treatment

## Abstract

**Rationale:**

Patients with relapsed and refractory Philadelphia chromosome-positive acute lymphoblastic leukemia (Ph+ ALL) with the T315I mutation are at higher risk of relapse and have shorter overall survival.

**Patient concerns:**

A 31-year-old man presented to the hematology department with intermittent fever and pancytopenia. He was diagnosed with Ph+ acute lymphoblastic leukemia and experienced 2 relapses during treatment. A drug-resistant T315I mutation was detected in the ABL kinase region during review.

**Diagnoses:**

Morphological examination of the bone marrow revealed approximately 93.5% lymphoid blast. Flow cytometric analysis confirmed the diagnosis of common B-cell ALL with the following phenotype: CD34, CD45dim, CD19, CD10, cCD79a, CD58dim, CD81dim, cTdT, HLA-DR, CD22dim, CXCR4, CD33dim, CD20, CD25, CD13, CD123. The examination of the ABL kinase region mutation suggested a T315I mutation.

**Interventions:**

Olverembatinib, a third-generation TKI drug, was administered in combination with inotuzumab ozogamicin to treat the disease.

**Outcomes:**

The patient achieved morphological remission with a negative flow cytometry MRD test, and the quantification of BCR-ABL transcripts was 0% after 1 cycle of therapy.

**Lessons:**

The third-generation TKI olverembatinib has been proven to be effective in CML patients with the T315I mutation, and it may also be effective in Ph+ acute lymphoblastic leukemia. Some new immune drugs have also shown improvement in the remission rate. Combination therapy with olverembatinib and Ino can achieve a complete molecular response in patients with relapsed and refractory Ph+ ALL with the T315I mutation.

## 1. Introduction

The Philadelphia chromosome positive acute lymphoblastic leukemia carries poor prognosis. The morbidity is about 20% to 25% in adult, and the incidence rate increases by age.^[1,2]^ The Philadelphia chromosome is formed by a translocation of the ABL1 gene on chromosome 9 and the BCR gene on chromosome 22. The fusion gene BCR-ABL can encode either p210 or p190 protein, thus causing excessive proliferation of malignant cells. The tyrosine kinase inhibitor (TKI) developed on this basis has shown superior effects. However, the first- and second-generation of TKI drug combined with chemotherapy as a standard regimen still has a high rate of relapse, and the relapse is often accompanied by drug-resistance BCR-ABL1 mutation, especially T315I mutation, which is related to highly aggressive disease phenotype and poor prognosis.^[3]^ Therefore, hematopoietic stem cell transplantation is still recommended for patients whose disease is under control. Besides, some new drugs, such as third-generation TKI drug ponatinib, antibody-drug conjugate, Bispecific Monoclonal Antibodies and CART, have provided new therapeutic strategies for Ph+ acute lymphoblastic leukemia. For example, inotuzumab ozogamicin or blinatumomab in combination with TKI drugs have been used in some relapsed refractory patients and deep molecular remission has been achieved within 3 months.^[4,5]^

In recent years, the third-generation TKI drug olverembatinib are recommended f or patients with the *BCR-ABL1^T315I^* mutation, which has achieved good curative effect in chronic and accelerated phase of chronic myeloid leukemia.^[6]^ Inotuzumab ozogamicin, as an antibody-drug conjugated which target CD22 on cell surface, has been proven to have a higher response rate than standard chemotherapy. So, inotuzumab ozogamicin plus olverembatinib has been used in this case and deep molecular remission has been achieved.

## 2. Case report

Our case is a 31-year-old male who was referred to our hospital in February 2022 with symptoms of “intermittent fever” and abnormal complete blood cell with white blood cell 370.3 × 10^9^/L, hemoglobin 51 g/L and platelets 25 × 10^9^/L. Morphological examination of bone marrow showed about 93.5% lymphoid blast. Flow cytometric analysis demonstrated the diagnosis of common B-cell ALL with a phenotype of CD34, CD45^dim^, CD19, CD10, cCD79a, CD58^dim^, CD81^dim^, cTdT, HLA-DR, CD22^dim^, CXCR4, CD33^dim^, CD20, CD25, CD13, CD123. Karyotype analysis showed that all of the 20 mitotic karyotype were abnormal (46,XY, t(4;12)(q31;q13), der(7), t(7;9)(p13;q22), t(9;22)(q34;q11.2), −7, −9, del(20)(q11.2), der(22)t(9;22), +der(22)t(9;22), +mar[20]). And quantitative Real-time PCR also showed that quantitative of BCR-ABL(P210) transcripts had achieved 113.44%. The patient had no history of chronic myelocytic leukemia and the spleen was not enlarged.

On February 2022, VCP (cyclophosphamide 750 mg/m^2^, D1, 15; vincristine 4 mg, D1, 8, 15, 22; prednisone acetate 60 mg D1–14; 40 mg D15–22) plus imatinib mesylate (IM) 400 mg QD were administered. Morphological remission was achieved after 1 course of treatment, and the quantification of BCR/ABL1 transcripts was 11.14%. The second course chemotherapy was not regular administrated due to the influence of COVID-19 pneumonia. During this period, patients just received oral imatinib 400 mg daily for 2 months. In May, Morphological examination of bone marrow showed about 67% lymphoid blast indicating hemotological relapse. Besides, the E255M mutation was found in the ABL kinase region and the BCR-ABL/ABL transcripts (P210) level was 103.34%. Therefore, We treated with Hyper-CVAD (cyclophosphamide 300 mg/m^2^, D1–3; vincristine 4 mg, D4,D11; adriamycin 75 mg/m^2^ D4, dexamethasone 40 mg D1–4, D11–14) and changed the TKI drug to dasatinib 100 mg QD oral administration, hematological remission was achieved on 21 days of treatment. After that, the patient continued to use dasatinib orally without chemotherapy for 3 weeks, and bone marrow morphology examination indicates recurrence with 70% blast cell and BCR-ABL/ABL transcripts level was 87.67%. Besides, the ABL kinase region mutation examination suggested a T315I mutation. The patient did not want to undergo hematopoietic stem cell transplantation and there was no suitable donor available. So we tried to use Ino in combination with olverembatinib (Ino 0.8 mg/m^2^ d1, 0.5 mg/m^2^ d8, d15, olverembatinib 40 mg QOD) for salvage therapy. The patient achieved morphological remission with negative flow cytometry MRD test, and the quantification of BCR-ABL transcripts was 0% after 1 cycle therapy. However, leukemia cells were found in cerebrospinal fluid exfoliated cells and flow cytometry analysis showed 25.11% blast cells. Thus, the diagnosis of CNS leukemia was confirmed. So the intensive lumbar puncture and intrathecal injection treatment was given. Both flow cytometry and exfoliated cells were negative after 2 times of intrathecal injection, and the patient continued to receive intracranial treatment according to our department protocol.

In 1st August, 23 day after the initiation administration of CD22 Monoclonal antibody and olverembatinib therapy, the increasing of liver enzymes was registered with AST-3613.0 IU/L (range 5–50 U/L ), ALT-2308.7 U/L (range 15–40 U/L), ALP-109.6 U/L (range 45–125 U/L), GGT 206.8 U/L (range 10–60 U/L), Total bilirubin 31.2 µmol/L (range 0–26 µmol/L), Direct bilirubin 12.7 µmol/L (range 0–6.8 µmol/L). There were no positive indicators of hepatitis virus and autoimmune hepatitis could be found. The abdominal macrovascular ultrasound and liver ultrasound indicated thinning of the hepatic vein lumen and enlarged liver shadow. Liver morphology did not exclude hepatic small vein obstruction syndrome (VOD). In this case, signs of abdominal distention, jaundice and ascites associated with VOD as well as VOD imaging were present. Besides, other diseases associated with these signs were also excluded. So we confirmed that the diagnosis of VOD is clear. And we considered to be alert to drug-related liver injury caused by CD22 monoclonal antibody plus olverembatinib. Therefore, TKI drug and inotuzumab ozogamicin were suspended. We gave the patient symptomatic treatment with applying Steroid (methylprednisolone 60 mg QD and reducing dose to 40 mg when liver function improves) and hepatoprotectors to reduce transaminases. After 17 days, liver function gradually improved(transaminases decreased to within 2 times), which ursodeoxycholic acid 500 mg QD was continuously applied during this period. Chemotherapy with VCP regimen combined with olverembatinib was given as sequential treatment. Then the 3 cycle of hyper-CVAD sequential with high dose methotrexate plus cytarabine was followed and olverembatinib was sustained. Bone marrow morphology suggests continued CR. The BCR-ABL/ABL transcripts (P210) level was 0%. No abnormal leukemic cells were found in cerebrospinal fluid flow residual and exfoliated cells. And there was no recurrence of liver function abnormal.

## 3. Discussion

The case represents a R/R Ph+ ALL, with unfavorable T315I mutation, in which the possibility of using Ino in combination with olverembatinib to achieve CMR was discussed.

The prognosis of Ph+ ALL has gradually improved from the era of single chemotherapy to hematopoietic stem cell transplantation (HSCT), and significantly improved after the appearance of TKI drugs. However drug-resistance mutations were often found in people who used first- or second-generation TKI drugs, leading to reduced CMR rate, increased recurrence rate and poor outcome. Therefore, the new generation of TKI drug ponatinib was invented, which can overcome the drug-resistant T315I mutation to some extent. In a phase II study of ponatinib in Ph+ leukemias, the overall survival rate at a year in patients with Ph+ ALL was estimated to be 40% (median, 8 months), the rate of progression-free survival was estimated to be 7% (median, 3 months.^[7,8]^ Ponatinib has shown anti-leukemic activity and played a significant role in clinical treatment. However, patients treated with ponatinib were prone to have higher risk of cardiovascular events, serious fatal arterial occlusions or venous thrombosis events and were once asked by the US Food and Drug Administration (FDA) to suspend to sale.^[9,10]^ Furthermore, ponatinib is not yet available in the Chinese mainland. Therefore, It is necessary to develop a well-tolerated TKI drug. The third-generation TKI drug, olverembatinib, has shown good efficacy in CML for people who are resistant to first or second-generation TKI drugs and have T315I mutations. It has shown overall better safety profile, especially for arterial thrombotic events (AOE) which frequently occur in ponatinib, and has been better tolerated than ponatinib.^[11]^ Secondly, olverembatinib has a good effect on both BCR-ABL wild type and multiple mutations. It has a highly specific targeting effect on T315I mutations over ponatinib.

In addition, it has shown similar remission rates and prognosis to ponatinib in CML patients. In view of the superior efficacy of olverembatinib in CML, we have attempted to apply olverembatinib to Ph+ ALL patients with T315I mutations in the hope that similar efficacy can be achieved.

The literatures reported that HSCT had been an irreplaceable option to achieve long survival for relapsed Ph ALL. However, CR and MRD negativity were prerequisite for successful transplantation, so many patients are difficult to qualify for transplantation and have poor long-term survival rates. The remission rate of chemotherapy in combination with ponatinib in relapsed Ph+ ALL were not satisfactory. Olverembatinib in combination with chemotherapy had not been reported in ALL. Therefore more effective regimens had to be sought. To address this situation, some studies had attempted to incorporate immunotherapeutic approaches. Surprisingly immunotherapy had shown high remission rates in recent years in B-cell ALL. In an INO-VATE study, no matter in Ph+ ALL or Ph−ALL, INO was found to increase remission rates and achieve MRD-negativity comparing with standard chemotherapy, increasing the likelihood of bridging HSCT in patients.^[12]^ TOWER and INO-VATE Study on the treatment of relapsed/refractory acute lymphoblastic leukemia suggested that the tumor burden had little impact on the response to Ino treatment. Similarly, the other studies also demonstrated that the blinatumomab has high remission rates and longer overall survival than standard chemotherapy for R/R B-ALL. In the phase III study of the Tower clinical trial with Ph-ALL, median overall survival was longer in the blinatumomab group than chemotherapy group (7.7 vs 4 months).^[13]^ It is easy to see that new immunotherapeutic drugs inotuzumab ozogamicin and blinatumomab has improved patients the rate of complete remission on a chemotherapy-free way.

Furthermore, combination of TKI drugs and immunotherapy also improved remission rates and the rate of minimal residual disease (MRD)-negative. Compared with the INO-VATE Ph+ ALL trail, the rate of CR/CRi were 83% in inotuzumab ozogamicin with bosutinib.^[14]^ The combination of ponatinib and blinatumomab resulted in a complete molecular remission rate of 87%.^[15]^ Some studies indicate that MRD-negativity could be achieved with blinatumomab in combination with TKI drugs in non-MRD negative patients.^[16]^ Therefore, Early complete remission with novel immunotherapy offers the possibility of bridging hematopoietic stem cell transplantation. However, The patient had a high proportion of primitive cells before salvage treatment. Subgroup analysis found that among relapsed/refractory ALL patients treated with blinatumomab, the overall response rate was 73% in the group with bone marrow primitive cell proportion < 50%, while the group with bone marrow primitive cell proportion ≥ 50% had an overall response rate of only 29%. Therefore, blinatumomab treatment was not used for the patient.

We combined olverembatinib with Ino to treat R/R Ph+ ALL patients and achieved deep molecular remission. Although adverse events of VOD occurred during the process, the liver function improved significantly after we stopped using Ino. Hepatic veno-occlusive disease is commonly seen after strong chemotherapy, hematopoietic stem cell transplantation and Ino with hyperbilirubinemia, ascites, painful hepatomegaly and weight gain as the main signs. In this case, elevated transaminases and bilirubin are the main indicators along with peritoneal effusion and imaging abnormalities. However, the patient’s state and various indexes improved quickly with the treatment of hepatoprotective drugs, enzyme inhibitors and glucocorticoids. We analyzed the reasons for the occurrence of VOD, considering that it might be adverse drug reactions.^[17]^ In the INO-VATE phase III study, Ino has been shown to be more prone to hepatotoxic damage than standard chemotherapy (SC). Among the recruited patients, the incidence rate of VOD was 2.1% to the SC group and up to 14% in the Ino group.^[18]^ It is uncertain whether the combination therapy we used increases the risk of VOD or not. But when we again to use olverembatinib in combination with chemotherapy, the patient’s liver function improved compared to previous and no obvious abnormalities were found in liver imaging. Olverembatinib has not shown abnormal liver function in CML, nor has it been reported in ALL. The VOD side effects of CD22 monoclonal antibody were not exacerbated by olverembatinib. Ino in combination with bosutinib or ponatinib also did not show an increase in VOD with TKI drugs.

In addition, the patient had a central nervous system leukemia during our treatment, suggesting that olverembatinib and inotuzumab ozogamicin may do not be able to cross the blood–brain barrier to have a good preventive effect. The treatment of extramedullary disease with CD22 monoclonal antibody has not been studied, so the prevention of central nervous system leukemia should be strengthened. Due to the VOD and lacking of transplant donors, the patient has not yet undergone hematopoietic stem cell transplantation. In order to prevent the aggravation of VOD after transplantation, transplantation should be performed after the improvement of various indexes. In a word, the chemotherapy-free regimen of Inotuzumab plus olverembatinib offers the possibility to achieve molecular remission for patients with R/R Ph+ ALL. But attention still needs to be paid to preventing VOD and CNS leukemia. It is recommended to choose patients with no previous liver damage, no viral hepatitis and other potential liver damage factors when using this regimen. The effectiveness and safety of olverembatinib still need to be further explored and improved. We hope that this regimen will lead to a good prognosis for more patients.

## Acknowledgments

Tong Liu and Chang Wang diagnosed the patient and wrote manuscript; Yanping Yang and Yu Fu treated the patient; Yehui Tan reviewed the manuscript. Informed consent has been obtained from patients in the study.

## Author contributions

**Investigation:** Yan-ping Yang.

**Resources:** Yu Fu.

**Writing – original draft:** Tong Liu, Chang Wang.

**Writing – review & editing:** Ye-hui Tan.

## References

[1] Mancini M Scappaticci D Cimino G. A comprehensive genetic classification of adult acute lymphoblastic leukemia (ALL): analysis of the GIMEMA 0496 protocol. Blood. 2005;105:3434–41. 15650057 10.1182/blood-2004-07-2922

[2] Burmeister T Schwartz S Bartram CR Gökbuget N Hoelzer D Thiel E; GMALL study group. Patients’ age and BCR-ABL frequency in adult B-precursor ALL: a retrospective analysis from the GMALL study group. Blood. 2008;112:918–9. 10.1182/blood-2008-04-149286 18650471

[3] Soverini S Iacobucci I Baccarani M Martinelli G. Targeted therapy and the T315I mutation in Philadelphia-positive leukemias. Haematologica. 2007;92:437–9. 17488653 10.3324/haematol.11248

[4] Ueda T Fukushima K Kusakabe S. Inotuzumab ozogamicin and blinatumomab sequential therapy for relapsed/refractory Philadelphia chromosome-positive acute lymphoblastic leukemia. Leuk Res Rep. 2022;17:100294. 35242526 10.1016/j.lrr.2022.100294 PMC8866140

[5] Stock W Martinelli G Stelljes M. Efficacy of inotuzumab ozogamicin in patients with Philadelphia chromosome-positive relapsed/refractory acute lymphoblastic leukemia. Cancer. 2021;127:905–13. 33231879 10.1002/cncr.33321 PMC7983935

[6] Dhillon S. Olverembatinib: first approval. Drugs. 2022;82:469–75. 35195876 10.1007/s40265-022-01680-9

[7] Cortes JE Kim DW Pinilla-Ibarz J. PACE Investigators. A phase 2 trial of ponatinib in Philadelphia chromosome-positive leukemias. N Engl J Med. 2013;369:1783–96. 24180494 10.1056/NEJMoa1306494 PMC3886799

[8] Cortes J Apperley J Lomaia E. Ponatinib dose-ranging study in chronic-phase chronic myeloid leukemia: a randomized, open-label phase 2 clinical trial. Blood. 2021;138:2042–50. 34407543 10.1182/blood.2021012082 PMC9728404

[9] Kantarjian HM DeAngelo DJ Advani AS. Hepatic adverse event profile of inotuzumab ozogamicin in adult patients with relapsed or refractory acute lymphoblastic leukaemia: results from the open-label, randomised, phase 3 INO-VATE study. Lancet Haematol. 2017;4:e387–98. 28687420 10.1016/S2352-3026(17)30103-5

[10] Cortes JE Kim DW Pinilla-Ibarz J. Ponatinib efficacy and safety in Philadelphia chromosome-positive leukemia: final 5-year results of the phase 2 PACE trial. Blood. 2018;132:393–404. 29567798 10.1182/blood-2016-09-739086 PMC6071555

[11] Jiang Q Li Z Qin Y. Olverembatinib (HQP1351), a well-tolerated and effective tyrosine kinase inhibitor for patients with T315I-mutated chronic myeloid leukemia: results of an open-label, multicenter phase 1/2 trial. J Hematol Oncol. 2022;15:113. 35982483 10.1186/s13045-022-01334-z PMC9389804

[12] Kantarjian HM Stock W Cassaday RD. Inotuzumab ozogamicin for relapsed/refractory acute lymphoblastic leukemia in the INO-VATE Trial: CD22 pharmacodynamics, efficacy, and safety by baseline CD22. Clin Cancer Res. 2021;27:2742–54. 33602684 10.1158/1078-0432.CCR-20-2399

[13] Kantarjian H Stein A Gökbuget N. Blinatumomab versus chemotherapy for advanced acute lymphoblastic leukemia. N Engl J Med. 2017;376:836–47. 28249141 10.1056/NEJMoa1609783 PMC5881572

[14] Jain N Maiti A Ravandi F. Inotuzumab ozogamicin with bosutinib for relapsed or refractory Philadelphia chromosome positive acute lymphoblastic leukemia or lymphoid blast phase of chronic myeloid leukemia. Am J Hematol. 2021;96:1000–7. 33991360 10.1002/ajh.26238 PMC9096877

[15] Jabbour E Short NJ Jain N. Ponatinib and blinatumomab for Philadelphia chromosome-positive acute lymphoblastic leukaemia: a US, single-centre, single-arm, phase 2 trial. Lancet Haematol. 2023;10:e24–34. 36402146 10.1016/S2352-3026(22)00319-2

[16] Curran E Stock W. Taking a “BiTE out of ALL”: blinatumomab approval for MRD-positive ALL. Blood. 2019;133:1715–9. 30796026 10.1182/blood-2018-12-852376 PMC6634959

[17] Mavrikou I Chatzidimitriou D Skoura L Nikolousis E Sakellari I Gavriilaki E. Molecular advances in sinusoidal obstruction syndrome/veno-occlusive disease. Int J Mol Sci . 2023;24:5620. 36982695 10.3390/ijms24065620 PMC10051970

[18] Kantarjian HM DeAngelo DJ Stelljes M. Inotuzumab ozogamicin versus standard of care in relapsed or refractory acute lymphoblastic leukemia: Final report and long-term survival follow-up from the randomized, phase 3 INO-VATE study. Cancer. 2019;125:2474–87. 30920645 10.1002/cncr.32116 PMC6618133

